# Supplementary material for: Sucrose rinse modulates the salivary behavior of carbonic anhydrase VI and its buffering capacity: a longitudinal study in 4 to 6.5-year-old children
Source: PeerJ. 2024 May 30;12:e17429. doi: 10.7717/peerj.17429 (PMC11144396; doi:10.7717/peerj.17429)
Supplement: Supplemental Information 2 [file peerj-12-17429-s002.docx]

**Supplementary Material**

**Simple effects for salivary flow rate**

*Disease*

| Time | Rinse | Sig. | | Partial Eta Squared | | Observed Power | |
| --- | --- | --- | --- | --- | --- | --- | --- |
| Baseline | Pre-rinse | .779 | .012 | | .087 | |  |
|  | Post-rinse | .367 | .046 | | .218 | |  |
| Follow-up | Pre-rinse | .314 | .052 | | .247 | |  |
|  | Post-rinse | .028 | .153 | | .672 | |  |

*Time*

| Disease | Rinse | Sig. | | Partial Eta Squared | | Observed Power | |  |
| --- | --- | --- | --- | --- | --- | --- | --- | --- |
| CF | Pre-rinse | | .006 | | .164 | | .811 | |
|  | Post-rinse | | .380 | | .018 | | .140 | |
| AC | Pre-rinse | | <.001 | | .248 | | .957 | |
|  | Post-rinse | | <.001 | | .287 | | .982 | |
| ACL | Pre-rinse | | .054 | | .084 | | .491 | |
|  | Post-rinse | | .351 | | .020 | | .152 | |

*Rinse*

| Disease | Time | Sig. | | Partial Eta Squared | | Observed Power | |  |
| --- | --- | --- | --- | --- | --- | --- | --- | --- |
| CF | Baseline | | <.001 | | .229 | | .937 | |
|  | Follow-up | | .835 | | .001 | | .055 | |
| AC | Baseline | | .002 | | .202 | | .897 | |
|  | Follow-up | | <.001 | | .249 | | .958 | |
| ACL | Baseline | | .041 | | .093 | | .538 | |
|  | Follow-up | | .707 | | .003 | | .066 | |

**Simple effects for buffering capacity**

*Disease*

| Time | Rinse | Sig. | | Partial Eta Squared | | Observed Power | |
| --- | --- | --- | --- | --- | --- | --- | --- |
| Baseline | Pre-rinse | .828 | .009 | | .078 | |  |
|  | Post-rinse | .590 | .024 | | .132 | |  |
| Follow-up | Pre-rinse | .745 | .014 | | .094 | |  |
|  | Post-rinse | .699 | .017 | | .104 | |  |

*Time*

| Disease | Rinse | Sig. | | Partial Eta Squared | | Observed Power | |  |
| --- | --- | --- | --- | --- | --- | --- | --- | --- |
| CF | Pre-rinse | | <.001 | | .253 | | .962 | |
|  | Post-rinse | | <.001 | | .362 | | .998 | |
| AC | Pre-rinse | | <.001 | | .236 | | .946 | |
|  | Post-rinse | | <.001 | | .327 | | .994 | |
| ACL | Pre-rinse | | <.001 | | .359 | | .998 | |
|  | Post-rinse | | <.001 | | .480 | | 1.000 | |

*Rinse*

| Disease | Time | Sig. | | Partial Eta Squared | | Observed Power | |  |
| --- | --- | --- | --- | --- | --- | --- | --- | --- |
| CF | Baseline | | .012 | | .138 | | .727 | |
|  | Follow-up | | .017 | | .126 | | .683 | |
| AC | Baseline | | .001 | | .223 | | .930 | |
|  | Follow-up | | <.001 | | .249 | | .958 | |
| ACL | Baseline | | <.001 | | .285 | | .982 | |
|  | Follow-up | | <.001 | | .290 | | .984 | |

**Simple effects for carbonic anhydrase VI**

*Disease*

| Time | Rinse | Sig. | Partial Eta Squared | Observed Power |
| --- | --- | --- | --- | --- |
| Baseline | Pre-rinse | .240 | .064 | .298 |
|  | Post-rinse | .536 | .029 | .149 |
| Follow-up | Pre-rinse | .924 | .004 | .061 |
|  | Post-rinse | .222 | .068 | .313 |

*Time*

| Disease | Rinse | Sig. | | Partial Eta Squared | | Observed Power | |  |
| --- | --- | --- | --- | --- | --- | --- | --- | --- |
| CF | Pre-rinse | | .020 | | .120 | | .659 | |
|  | Post-rinse | | .295 | | .025 | | .179 | |
| AC | Pre-rinse | | .023 | | .114 | | .634 | |
|  | Post-rinse | | .175 | | .042 | | .271 | |
| ACL | Pre-rinse | | .478 | | .012 | | .108 | |
|  | Post-rinse | | .386 | | .018 | | .137 | |

*Rinse*

| Disease | Time | Sig. | | Partial Eta Squared | | Observed Power | |  |
| --- | --- | --- | --- | --- | --- | --- | --- | --- |
| CF | Baseline | | <.001 | | .271 | | .974 | |
|  | Follow-up | | .314 | | .024 | | .169 | |
| AC | Baseline | | <.001 | | .348 | | .997 | |
|  | Follow-up | | .053 | | .085 | | .495 | |
| ACL | Baseline | | .202 | | .038 | | .245 | |
|  | Follow-up | | .354 | | .020 | | .150 | |
